# Evaluation of the applicability of deep breathing test in the diagnosis of hypertension with white-coat effect in Chinese patients in primary care

**DOI:** 10.1186/s40885-018-0106-4

**Published:** 2019-02-01

**Authors:** Kam Sum Chan, Kit Ping Loretta Lai, Pang Fai Chan, Man Hei Matthew Luk, Vai Kiong David Chao

**Affiliations:** Department of Family Medicine and Primary Health Care, United Christian Hospital and Tseung Kwan O Hospital, Kowloon East Cluster, Hospital Authority, Tseung Kwan O, Hong Kong, China

**Keywords:** White-coat effect, Deep breathing test, Hypertension, Primary care

## Abstract

**Purpose:**

The current gold standard for the diagnosis of white-coat effect is by the 24-h ambulatory blood pressure monitoring (ABPM) which may not be readily available in every primary care setting. Previous studies had shown that deep breathing, through modulating the baroreceptor reflex sensitivity to vagal stimulation over 30 to 60 s, was useful in detection of the white-coat effect. The aim of our study was to evaluate the diagnostic accuracy of the deep breathing test (DBT) as compared with the gold standard of ABPM in the diagnosis of hypertension with white-coat effect in Chinese patients in primary care.

**Methods:**

This cross sectional study recruited 178 consecutive, eligible, consented, hypertensive patients receiving treatment at a local public primary care Hypertension Clinic.

The diagnostic accuracy of the DBT in all recruited patients, patients not taking beta-adrenergic blockers and patients with different clinic SBP cut-off before the DBT by means of area under the receiver operating characteristic (ROC) curve, sensitivity, specificity, positive and negative predictive values was evaluated.

**Results:**

The results for the ROC curves for systolic and diastolic BP changes after the DBT were statistically insignificant. The ROC curve was statistically significant for SBP change in patients not taking beta-adrenergic blockers and with pre-DBT clinic SBP ≥ 165 mmHg (ROC curve area of 0.719, 95% CI 0.53 to 0.91, *p* = 0.04). The corresponding sensitivity and specificity of the DBT were 40.9 and 90.9% respectively if SBP drop was > 30 mmHg.

**Conclusion:**

The DBT, even though could not be clinically applied to all patients, was proven to be a potential screening and diagnostic test for white-coat effect in Chinese hypertensive patients with a pre-test SBP of ≥165 mmHg and who were not taking beta-adrenergic blockers.

**Trial registration:**

This study was approved by Kowloon East Cluster/ Kowloon Central Cluster Research Ethics Committee/Institutional Review Board of Hospital Authority of Hong Kong under the registration KC/KE-16-0084/ER-3.

## Introduction

Hypertension, being a major constituent to the global burden of non-communicable diseases [1], was reported to be prevalent in around 32% of the Hong Kong population in a local large cohort study in 2012 [2]. It is a leading risk factor of cardiovascular diseases which cause significant morbidity and mortality [3, 4]. However, only about half of the treated hypertensive patients achieved target blood pressures according to international studies [5, 6]. The reasons accounting for suboptimal blood pressure control included inadequate treatment, poor patient adherence, white-coat hypertension, undiagnosed secondary hypertension and true resistant hypertension [7, 8].

White-coat hypertension is defined as persistently raised office blood pressure in ambulatory normotensive patients [9]. It accounts for up to 20 to 30% of patients worldwide [10–12]. White-coat effect refers to a patient with home or ambulatory blood pressure within the hypertensive range but who exhibit a disproportionately raised clinic blood pressure reading [9]. It was reported to account for one in four of the treated hypertensive patients with suboptimal office blood pressure control [13, 14] and prevalent among 35–73% of patients with treated hypertension [12, 15–19]. Factors associated with white-coat hypertension including female sex, white ethnicity, increasing age, higher body mass index, higher clinic systolic blood pressure and declining renal function [12, 20, 21].

The detection of white-coat effect is important as failure to recognise the condition may lead to unnecessary treatment causing undesirable side effects for the patients and also inflating the cost of treatment [22–24]. Current gold standard for the diagnosis of white-coat effect is by the 24-h ambulatory blood pressure monitoring (ABPM) which can provide information about blood pressure during daily activities and sleep and thus better evaluation of white-coat effect than home blood pressure [9, 25, 26]. However, ABPM requires specific equipment and expertise to operate and may not be available in every primary care setting.

Deep breathing was shown to be able to lower blood pressure by increasing the baroreceptor reflex sensitivity to vagal stimulation [27, 28]. Studies had shown that deep breathing over 30 to 60 s was useful in detection of the white-coat effect by measuring the difference in systolic blood pressure (SBP) after performing the deep-breathing test (DBT) [29–32]. In the Federico et al. study, the DBT resulted in a statistically significant difference in mean SBP drop of 17.8 and 10.9 mmHg (*p* < 0.001) among patients with or without white-coat effect respectively [29]. In the Marion et al. study, a 15% drop in SBP was found to be corresponded to a 96% specificity (95% CI 79.0–100.0) and 94% positive predictive value (95% CI 72.0–100.0) in the diagnosis of white-coat hypertension [30]. The Jose et al. study evaluated the diagnostic accuracy of DBT on identifying white-coat hypertension. The study adopted two criteria as a positive response to DBT: a 10 mmHg drop in diastolic blood pressure (DBP) or normalisation of SBP to < 140 mmHg and DBP < 90 mmHg. They found no significant difference (*p* < 0.26) between the hypertensive patients with or without white-coat effect when using the first criterion but was able to identify a significant difference (*p* < 0.003) using the second criterion when applied to patients with office SBP < 160/100 mmHg [31]. The application of DBP drop in predicting white-coat hypertension was only found useful in the Yoshihara et al. study [32].

Previous evidence also supported a positive correlation between office SBP and white-coat effect [20, 21]. In a Taiwanese study which compared the characteristics of patients with or without white-coat effect as defined by the gold standard 24 h ABPM, office SBP was found to be significantly correlated with white-coat effect (odd ratio 1.079, 95% CI 1.034–1.125, *p* < 0.001) [20]. In another large scale study involving over 2000 patients in Greece, a 1.0 mmHg increase in daytime SBP variability was correlated with an increase of 0.589 mmHg [95% CI 0.437–0.741] in the systolic white coat effect [21].

There was also evidence showing that beta-adrenergic blockers might potentiate the baroreflex, possibly through enhancing heart-rate variability and increasing the vagal tone while reducing the sympathetic beta-receptor stimulation and therefore might affect the DBT results [33, 34]. However, the effect of beta-adrenergic blockers on DBT was not considered in all the previous mentioned studies in detecting white-coat effect. To our knowledge, all of the current evidence of clinical application of DBT was derived from non-Chinese population. There was a lack of research data on the validity of DBT in detecting white-coat effect within the Chinese hypertensive population. The effects of different clinic SBP and the use of beta-adrenergic blockers on the application of DBT in Chinese were also lacking. This study therefore aimed to evaluate the applicability of DBT as an alternative diagnostic test of white-coat effect, which would be much less time and resource consuming as compared with the 24-h ABPM in Chinese hypertensive patients. Sub-analysis on the applicability of DBT in detecting white-coat effect in patients with different clinic SBP cut-off and patients not taking beta-adrenergic blockers would also be performed.

## Methodology

### Study design

This was a cross sectional study conducted in a public primary care clinic serving more than 10,000 hypertensive patients in year 2015. When patient failed to reach target clinic blood pressure during consecutive follow up visits, they would be asked to bring their home blood pressure machine for validation along with their home blood pressure record. If there was a significant discrepancy between the clinic blood pressure and home blood pressure as measured by a validated home blood pressure machine, they would be referred to the Hypertension Clinic for further evaluation by family medicine specialists. Consecutive patients on anti-hypertensives, attending the Hypertension Clinic and fulfilled the inclusion criteria i.e. with clinic SBP ≥ 140 mmHg and/or DBP ≥ 90 mmHg at latest two clinic visits during the study period were included. Only those who aged 18 years or above and agreed to give consent in participating in the study were recruited until the required sample size was reached. Non-Chinese patients, patients with suspected secondary hypertension or atrial fibrillation and pregnant patients were excluded. The study was conducted from 1st August 2016 to 30th September 2017. This study was approved by Kowloon East Cluster/ Kowloon Central Cluster Research Ethics Committee/Institutional Review Board of Hospital Authority of Hong Kong.

### Procedures

All clinic blood pressure readings were obtained in sitting position with the measuring cuff at heart level, using the calibrated manual sphygmomanometers (UM-101, A&D instruments Ltd., Abingdon, Oxon, U.K) and appropriate cuff sizes. The patient, after resting for at least 5 min, would have his or her blood pressure measured at both arms. The second blood pressure, spaced 2 min apart would then be taken at the arm with higher measured blood pressure [26]. The highest blood pressure values would be used as the clinic blood pressure before performing the DBT. During the DBT, the patient had to take deep breathing cycles for sixty seconds, around one cycle every ten seconds. The patient would be instructed to simulate the respiratory pattern based on an application of tempo counter installed in a smartphone. Another blood pressure would be measured immediately after the DBT on the previously chosen arm. All blood pressure measurements and DBTs would be performed by the same trained nurse blinded to the 24-h ABPM results.

Subsequently, a monitor (TM-2430, A&D instruments Ltd., Abingdon, Oxon, U.K) would be installed to perform the 24-h ABPM with an appropriate cuff placed on the non-dominant arm and programmed to measure blood pressure every 30 min while patients were awaked and 60 min while patients were asleep. The awake and sleeping time would be programmed individually based on the history given by patients. Patients with ABPM reports showing more than 80% successful readings would be included and their results would be interpreted by family medicine specialists who were blinded to the DBT results. There was no internationally standardized definition on the interpretation of white-coat effect by using the 24-h ABPM. In this study, it would be defined as a decrease of 10 mmHg or more in mean daytime ambulatory SBP when compared with the clinic SBP [9].

Patients’ demographic data including age, gender, body mass index, smoking status, presence of diabetes and cardiovascular diseases and number and types of anti-hypertensives taken were retrieved from computerized record.

### Sample size calculation

Previous studies showed that the sensitivity of using 10 mmHg difference as cut-off to define white-coat effect after DBT was 0.8 [29]. The prevalence of white-coat effect in hypertensive Chinese patients was lacking. Taking 35% as the most conservative estimated prevalence of white-coat effect in treated hypertensive patients in previous international studies [12, 15–19] and maximum marginal error of estimate as 0.10, the sample size needed was 176 [35].

### Outcome

The main outcome was to evaluate the diagnostic accuracy of the DBT for white-coat effect by means of area under the receiver operating characteristic (ROC) curve, sensitivity, specificity, positive and negative predictive values in Chinese hypertensive patients. Sub-analysis on the effect of beta-adrenergic blockers and different clinic SBP cut-off on the diagnostic accuracy of the DBT would also be evaluated. Blood pressure responses to DBT in all patients and different sub-groups with or without white-coat effect would be presented in terms of mean blood pressure drop after the test.

### Statistical analysis

Central tendencies and distributions of continuous variables were presented as means and standard deviations respectively. The means of continuous variables were compared with independent samples t test. Categorical variables were presented as proportions and percentages. They were compared with Chi-square test (with Yate’s correction for 2 × 2 comparisons) or Fisher’s exact test. Areas under the receiver operating characteristic (ROC) curve, sensitivity, specificity, positive and negative predictive values were calculated to assess the diagnostic accuracy of the DBT, with respective 95% confidence interval. Differences were considered statistically significant when *p* < 0.05. SPSS version 21 was used for statistical analysis.

## Results

### Study population

Five Hundred forty-eight patients attended the Hypertension Clinic during the study period and 209 patients fulfilled the inclusion criteria. 30 (14.4%) patients refused to participate and 1 patient was excluded due to insufficient ABPM data resulting in 178 patients being recruited into the study. The mean age of the subjects was 65.0 and more were female patients (64%). More than half of the subjects (54.5%) were receiving monotherapy. Calcium channel blockers were the commonest (74.2%) class of anti-hypertensives being used by the patients, followed by angiotensin-converting enzyme inhibitors (27.5%), beta-adrenergic blockers (21.3%), angiotensin receptor blockers (14.6%), alpha-blockers (9.0%), diuretics (8.4%), hydralazine (7.9%) and methyldopa (5.1%). Other clinical characteristics of the subjects were summarized in Table 1. The baseline characteristics including age, sex, body mass index, smoking status, presence of diabetes and cardiovascular disease showed no statistically significant difference between the white-coat and non white-coat groups.

**Table 1:** Demographic data and clinical characteristics of patients with white-coat and without white-coat effect (*N* = 178)

Clinical characteristics	White-coat effect		Total no.	*p* value
	Present (*n* = 122)	Absent (*n* = 56)	(%)	
Age (years)				
Mean	65.6 (SD 9.7)	63.8 (SD 10.7)	65.0 (SD 10)	0.270
No. of patients < 40	1	1	2 (1.1)	
40–49	8	4	12 (6.7)	
50–59	23	14	37 (20.8)	
60–69	48	19	67 (37.7)	0.791
70–79	34	16	50 (28.1)	
≥ 80	8	2	10 (5.6)	
Sex				
Male	45	19	64 (36.0)	0.703
Female	77	37	114 (64.0)	
Body mass index (kg/m^2^)				
Mean	25.3 (SD 3.7)	25.6 (SD 3.8)		0.618
No. of patients < 23	28	17	45 (25.3)	
23–24.9 (overweight)	36	11	47 (26.4)	0.317
≥ 25 (obesity)	58	28	86 (48.3)	
Smoking status				
Non smoker	101	43	144 (80.9)	0.296
Ex-smoker	19	13	32 (1.1)	
Current smoker	2	0	2 (18.0)	
Presence of diabetes				
Yes	38	17	55 (30.9)	0.916
No	84	39	123 (69.1)	
History of cardiovascular disease				
Yes	8	5	13 (7.3)	0.757
No	114	51	165 (92.7)	
No. of anti-hypertensives taken				
Monotherapy	66	31	97 (54.5)	
Two drugs	33	16	49 (27.5)	0.866
Three or more drugs	23	9	32 (18.0)	
Types of anti-hypertensives taken				
Alpha-blockers	9	7	16 (9.0)	0.267
Angiotensin-converting enzyme inhibitors	34	15	49 (27.5)	0.881
Angiotensin receptor blockers	17	9	26 (14.6)	0.820
Beta-adrenergic blockers	28	10	38 (21.3)	0.555
Calcium channel blockers	94	38	132 (74.2)	0.193
Diuretics	10	5	15 (8.4)	1
Hydralazine	8	5	14 (7.9)	0.757
Methyldopa	7	2	9 (5.1)	0.722

### Deep breathing test results

In all subjects, the DBT reduced SBP by a mean of 14.9 mmHg and DBP by a mean of 4.3 mmHg (*p* < 0.001) (Table 2). 68.5% (122 out of 178) of the subjects were found to have white-coat effect by ABPM. The mean SBP and DBP drop after the DBT for the white-coat effect group were 15.5 mmHg and 4.4 mmHg respectively while for the non white-coat effect group were 13.3 mmHg and 3.8 mmHg respectively.. Neither the SBP nor DBP drop showed a statistically significant difference. The areas under the ROC curve for SBP and DBP changes in all patients were 0.52 (95% CI 0.43–0.61, *p* = 0.611) and 0.53 (95% CI 0.44–0.62, *p* = 0.552) respectively, showing that they were uninformative (Table 3).

**Table 2:** BP responses to the deep breathing test in all patients (*N* = 178)

Variables	Mean BP before DBT (mmHg)	Mean BP after DBT (mmHg)	*p* value
Systolic BP	153.6 (SD 17.4)	138.7 (SD 16.9)	< 0.001
Diastolic BP	77.1 (SD 10.5)	72.8 (SD 10.7)	< 0.001

*BP* blood pressure, *DBT* deep breathing test

**Table 3:** Deep breathing test results in all patients (*N* = 178)

BP response to the deep breathing test	White-coat effect		*p* value
	Present (*n* = 122)	Absent (*n* = 56)	
Mean SBP drop (mmHg)	15.5 (SD 12.0)	13.3 (SD 11.7)	0.247
Mean DBP drop (mmHg)	4.4 (SD 5.2)	3.8 (SD 4.3)	0.436
Deep breathing test operating characteristics	Area under ROC curve	95% confidence interval (CI)	*p* value
SBP change	0.52	0.43–0.61	0.611
DBP change	0.53	0.44–0.62	0.552

*BP* blood pressure, *SBP* systolic blood pressure, *DBP* diastolic blood pressure, *DBT* deep breathing test, *ROC* Receiver operating characteristic

### Subgroup analysis with different pre-test SBP cut off

In the sub-analysis of patients with different clinic pre-test SBP cut-off, the mean SBP drop showed significant difference in patients with clinic SBP ≥145 mmHg before the DBT (*p* = 0.042). The ROC curve for both SBP and DBP change did not yield any significant results. However, the discriminating power of the DBT by using SBP change was improved with higher pre-test SBP cut-off, with an area under ROC curve of 0.68 (95% CI 0.51–0.84) and *p* value reaching 0.07 when patients with SBP < 165 mmHg excluded (Table 4).

**Table 4:** Sub-group analysis of deep breathing test results with different pre-test SBP cut-off

BP response to the deep breathing test	White-coat effect		*p* value
	Present	Absent	
(i) SBP ≥ 145 mmHg (*N* = 118)	*n* = 78	*n* = 40	
Mean SBP drop (mmHg)	19.2 (SD 12.8)	14.1 (SD 12.5)	*0.042
Mean DBP drop (mmHg)	4.9 (SD 5.4)	3.8 (SD 4.3)	0.268
(ii) SBP ≥ 155 mmHg (*N* = 74)	*n* = 51	*n* = 23	
Mean SBP drop (mmHg)	20.8 (SD 14.5)	15.1 (SD 13.0)	0.107
Mean DBP drop (mmHg)	4.8 (SD 6.3)	4.3 (SD 4.2)	0.739
(iii) SBP ≥ 165 mmHg (*N* = 43)	*n* = 30	*n* = 13	
Mean SBP drop (mmHg)	24.4 (SD 15.4)	15.3 (SD 13.2)	0.073
Mean DBP drop (mmHg)	4.9 (SD 5.8)	3.5 (SD 3.5)	0.451
Deep breathing test operating characteristics	Area under ROC curve	95% confidence interval (CI)	*p* value
(i) SBP ≥ 145 mmHg (N = 118)			
SBP change	0.60	0.49–0.70	0.087
DBP change	0.55	0.44–0.66	0.365
(ii) SBP ≥ 155 mmHg (N = 74)			
SBP change	0.61	0.48–0.74	0.130
DBP change	0.51	0.37–0.65	0.912
(iii) SBP ≥ 165 mmHg (N = 43)			
SBP change	0.68	0.51–0.84	0.070
DBP change	0.57	0.39–0.74	0.500

*BP* blood pressure, *SBP* systolic blood pressure, *DBP* diastolic blood pressure, *DBT* deep breathing test, *ROC* receiver operating characteristic

### Subgroup analysis with patients on beta-adrenergic blockers excluded

**Fig. 1:**
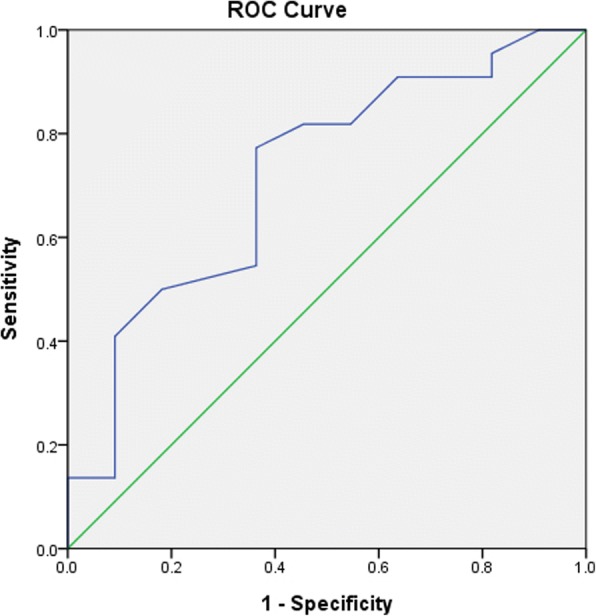
ROC curve in patients with SBP ≥165 mmHg showed that SBP change was a good diagnostic test for white-coat effect with the area under curve 0.72 (95% CI 0.53–0.91, *p* = 0.04)

23% of the patients with white-coat effect and 17.9% of the non white-coat effect group were taking beta-adrenergic blockers. The difference in mean SBP and DBP drop after the DBT between the white-coat and non white-coat groups in patients not on beta-adrenergic blockers was not statistically significant. However, when combining the two variables, with different pre-test SBP cut-off and the exclusion of patients taking beta-adrenergic blockers, the difference in mean SBP drop reached statistical significance in patients with SBP ≥145 mmHg (*p* = 0.043) and ≥ 165 mmHg (*p* = 0.035). (Table 5) On the other hand, the mean DBP drop did not show any statistically significant difference between the two groups by all means of the aforementioned analysis. Further analysis by means of the ROC curve (Table 5) showed that SBP change was a good diagnostic test for white-coat effect in patients with pre-test SBP ≥165 mmHg with an area under curve of 0.72 (95% CI 0.53–0.91, p = 0.04) (Fig. 1).

**Table 5:** Sub-analysis of deep breathing test results with exclusion of patients on beta-adrenergicblockers

BP response to the deep breathing test	White-coat effect		*p* value
	Present	Absent	
A. Patients not on beta-adrenergic blockers (*N* = 140)	*n* = 94	*n* = 46	
Mean SBP drop (mmHg)	15.7 (SD 11.5)	12.8 (SD 11.0)	0.149
Mean DBP drop (mmHg)	4.5 (SD 5.1)	3.9 (SD 4.3)	0.465
B. Patients not on beta-adrenergic blockers and with different clinic SBP cut-off before DBT			
(i) SBP ≥ 145 mmHg (*N* = 92)	*n* = 58	*n* = 34	
Mean SBP drop (mmHg)	19.8 (SD 12.1)	14.5 (SD 11.5)	*0.043
Mean DBP drop (mmHg)	4.9 (SD 5.2)	4.1 (SD 4.3)	0.449
(ii) SBP ≥ 155 mmHg (N = 58)	*n* = 38	*n* = 20	
Mean SBP drop (mmHg)	22.0 (SD 13.4)	15.1 (SD 13.7)	0.068
Mean DBP drop (mmHg)	4.9 (SD 6.0)	4.6 (SD 4.4)	0.847
(iii) SBP ≥ 165 mmHg (*N* = 33)	*n* = 22	n = 11	
Mean SBP drop (mmHg)	25.6 (SD 13.1)	14.6 (SD 14.3)	*0.035
Mean DBP drop (mmHg)	6.1 (SD 5.6)	3.8 (SD 3.6)	0.232
Deep breathing test operating characteristics	Area under ROC curve	95% confidence interval (CI)	p value
A. Patients not on beta-adrenergic blockers (*N* = 140)			
SBP change	0.56	0.46–0.66	0.288
DBP change	0.53	0.43–0.63	0.554
B. Patients not on beta-adrenergic blockers and with different clinic SBP cut-off before DBT			
(i) SBP ≥ 145 mmHg (*N* = 92)			
SBP change	0.62	0.50–0.73	0.066
DBP change	0.54	0.41–0.66	0.580
(ii) SBP ≥ 155 mmHg (*N* = 58)			
SBP change	0.64	0.49–0.78	0.094
DBP change	0.51	0.35–0.66	0.948
(iii) SBP ≥ 165 mmHg (*N* = 33)			
SBP change	0.72	0.53–0.91	*0.040
DBP change	0.62	0.42–0.82	0.260

*BP* blood pressure, *SBP* systolic blood pressure, *DBP* diastolic blood pressure, *DBT* deep breathing test, *ROC* receiver operating characteristic

The corresponding sensitivity, specificity, positive and negative predictive values for SBP drop in patients not on beta-adrenergic blockers and with pre-test SBP ≥165 mmHg were shown in Table 6. A drop of more than 10 mmHg in the SBP was highly sensitive (90.9%), however, the specificity was only 36.4%. If SBP drop was 30 mmHg or more, sensitivity decreased to 40.9% but specificity increased to 90.9%. The positive predictive value (PPV), ranging from 74 to 90%, was most predictive when the SBP drop was more than 30 mmHg. The negative predictive value (NPV) was less informative, with the highest value reaching 67% if SBP drop was more than 10 mmHg.

**Table 6:** Deep breathing test operating characteristics for SBP drop in patients not on beta-adrenergic blockers and with pre-test SBP ≥165 mmHg

Cut-off value	Sensitivity (%)	Specificity (%)	PPV (%)	NPV (%)
> 10 mmHg	90.9	36.4	74	67
> 20 mmHg	77.3	63.6	81	58
> 30 mmHg	40.9	90.9	90	43
> 40 mmHg	13.6	90.9	75	34

*SBP* systolic blood pressure, *PPV* positive predictive value, *NPV* negative predictive value

## Discussion

Even though ABPM remains the gold standard in the diagnosis of white-coat effect in hypertension, it requires specific equipment and extra manpower that might not be readily available in every primary care physician’s office. A much simpler DBT would be invaluable to detect white-coat effect and avoid unnecessary over-treatment.

Our study showed an important finding that DBT was useful in detecting white-coat effect in a specific group of Chinese hypertensive patients i.e. patients with pre-test SBP ≥165 mmHg and who were not taking beta-adrenergic blockers. In this group of patients, the DBT showed high specificity (90.9%) if the post-test SBP drop was more than 30 mmHg. The relatively high PPV and low NPV implied that DBT was most useful to rule in the existence of white-coat effect.

Our study was one of the studies with larger sample size as compared to previous studies and probably the first to examine the deep breathing test’s applicability in Chinese patients. Furthermore, our study was the first to have taken into consideration the practicality of performing the DBT in actual clinical setting where the patients were more likely to be continued on their usual drug regime while performing the DBT. Neither the previous studies conducted by Federico et al. and Marion et al. took into account for the types of anti-hypertensives the patients were taking while they were performing the DBT, while in the Jose et al. study, the study subjects had anti-hypertensive withdrawn for 2–3 weeks before attempting the DBT, which might be less practical in a non-research setting.

Our study added information that beta-adrenergic blockers and different clinic SBP cut-off would affect the DBT performance. Since higher office SBP was positively correlated with white-coat effect, we postulated that patients with higher pre-test SBP would exhibit significant difference in BP responses after DBT between the white-coat and non white-coat groups. Though the mean SBP drop between the two groups did not show increasing significance as we moved towards higher SBP cut-off, it reached statistical significance across both groups with SBP ≥145 mmHg and ≥ 165 mmHg and patients not on beta-adrenergic blockers. The findings also supported the evidence that beta-adrenergic blockers could modify the baroreflex and hence affect the DBT results.

Previous studies showed conflicting evidence on whether the drop of SBP or DBP post-DBT could be used to evaluate the presence of white-coat effect [29–32]. Our study showed that DBP drop was not useful in detecting white coat effect which was compatible with most of the other published studies [29–31]. .For SBP drop, our study did not reach a significant value when all patient analysis was performed. Ethnicity might have partly accounted for this difference. Another reason we postulated was the way that DBT was performed, which differed among studies. In the Marion et al. study, the study subjects were asked to perform 3 or 4 cycles of deep breathing in 30 s while in the Federico et al. study, the number of deep breathing cycles was not specified. Only in the Jose et al. study were the patients asked to perform DBT according to the analog clock who simulated 0.1 Hz breathing cycle. The advantage of which was a more consistent and reproducible test result and also more conforming to the original idea behind synchronising the breathing cycle with the innate cardiovascular rhythms to modulate the baroreflex.

### Limitation

Our study showed a significant result after exclusion of patients on beta-adrenergic blockers and those with pre-test SBP < 165 mmHg. However, the sample size by such exclusivity became smaller and hence affected the statistical accuracy to find a precise SBP cut-off for distinguishing between the white-coat and non white-coat effect. Further research is suggested to specifically investigate this sub-group with a larger sample size. Moreover, the study was conducted in only one single clinic which was specialized on assessing hypertensive patients with suspected white-coat effect. The clinic had exceptionally more patients with white-coat effect diagnosed (68.5%) and the results might not be able to be generalised to all primary care clinic settings in Hong Kong.

Throughout the study, there were several occasions where the post DBT blood pressures were higher than the pre-test values. As mentioned earlier, the device guided breathing cycle had its advantages, nonetheless, it also increased the anxiety in some of our patients during the attempt to synchronise their breathing rate to that of the counter on the mobile device. More training and attempts might be needed to allow the patients more adapt to the deep breathing exercise and produce a more pronounced blood pressure lowering effect. For those patients who showed a paradoxically increase in blood pressure after DBT, further evaluation with a standard ABPM should be performed.

## Conclusion

The DBT could not be clinically applied to all Chinese patients with suspected white-coat effect. However, the study proved that it would be a potential screening and diagnostic test for white-coat effect under certain selection criteria; i.e. in patients with a pre-test SBP of ≥165 mmHg and who were not taking beta-adrenergic blockers. Future larger scale studies should be conducted to enhance the statistical accuracy and generalisability of the results.

## Abbreviations

ABPM: Ambulatory blood pressure monitoring
BP: Blood pressure
CI: Confidence interval
DBP: Diastolic blood pressure
DBT: Deep breathing test
NPV: Negative predictive value
PPV: Positive predictive value
ROC: Receiver operating characteristic
SBP: Systolic blood pressure
